# An Investigation of Sensory Specific Satiety and Food Size When Children Consume a Whole or Diced Vegetable

**DOI:** 10.3390/foods6070055

**Published:** 2017-07-24

**Authors:** Jasmine R. Goh, Catherine G. Russell, Djin G. Liem

**Affiliations:** 1 Faculty of Health, Centre for Advanced Sensory Science, School of Exercise and Nutrition Sciences, Deakin University, 221 Burwood Highway, Burwood, VIC 3125, Australia; reena@bigpond.net.au (J.R.G.); georgie.russell@deakin.edu.au (C.G.R.); 2 Faculty of Health, University of Technology Sydney, Broadway 2007, PO Box 123, Sydney, Australia

**Keywords:** children, sensory specific satiety, carrot, vegetable, consumption, liking, taste, variety, unit bias

## Abstract

Children’s vegetable consumption is often lower than that needed to promote optimal health and development, and practical approaches for increasing vegetable consumption are needed. Sensory Specific Satiety (SSS) reduces the liking and consumption of a consumed food over the course of an eating occasion and is an important factor in meal termination. The present study aimed to investigate the development of SSS when children ate vegetables of different sizes. The absence of SSS would be an encouraging sign to provide children more vegetables during a meal. Seventy-two children (33 boys, ages 8.8 ± 1.5 years) were recruited from Australian primary schools. Participating children consumed either whole or diced carrots for a maximum period of 10-min from a 500 g box. Cucumber was used as a control vegetable. Children’s liking of carrots and cucumber was measured with a 5-point child friendly hedonic scale prior to and after carrot consumption. In comparison to cucumber, liking for neither diced (*p* = 0.57) nor whole carrots (*p* = 0.18) changed during ad libitum consumption of carrots, indicating that SSS did not occur. However, children (*n* = 36) who finished eating carrots within the 10-min time limit, spent more time eating the whole carrots compared to the diced carrots (*p* < 0.05), which tended to result in a higher consumption of whole carrots (*p* < 0.06). This suggests that, in order to increase vegetable consumption, it is better to present children whole carrots than diced carrots. These findings might aid in the development of strategies to promote children’s greater vegetable consumption.

## 1. Introduction

High vegetable consumption during childhood is important for children’s optimal health and development, and the prevention of obesity and associated chronic diseases later in life [1]. Childhood is a sensitive period in which children learn about food and eating [2,3,4], which has a lifelong impact upon dietary patterns [5,6]. It is important, then, that children consume adequate amounts and variety of vegetables during childhood to promote good longer-term health. The most recent national dietary data from Australia indicate that very few children actually consume the recommended amounts of vegetables (i.e., 5 serves of vegetables per day): less than 10% of children aged 4–18 years met the recommendations [7]. Similar patterns are observed in a range of other countries [8,9,10]. Strategies for increasing children’s vegetable consumption are therefore needed.

The reasons for children’s low consumption of vegetables are multifaceted [3,11,12] and a range of strategies is needed to increase consumption to the levels of those recommended by governments. One relatively simple approach to increasing vegetable intakes is to offer children (and infants) a greater variety of (liked to moderately liked) vegetables during a meal [13,14,15,16]. There is some evidence that by offering children a greater variety of vegetables, their total vegetable consumption during one sitting may increase, although findings are equivocal. Roe et al. [17], found that by offering preschool aged children a variety (3) of vegetable snacks in their childcare setting, children were more likely to select and consume vegetables even when they were not particularly liked. However, Zeinstra et al. found that offering children two moderately liked vegetables instead of one did not increase 4–6 year old children’s vegetable consumption in a restaurant setting [18]. A clear understanding of the mechanisms that may underlie children’s food intakes when greater variety is offered is needed to gain insight as to why variety might promote greater consumption in some instances and not others.

One explanation for a possible increased intake from a variety of foods being offered is Sensory Specific Satiety or lack thereof. Sensory Specific Satiety (SSS) is the change in liking of a food that has been consumed ad libitum relative to a change in liking of a food that has not been consumed ad libitum [19]. SSS has the effect of reducing liking and subsequently consumption of a particular food (or meal) during the course of eating it [19]. A range of SSS experiments has demonstrated the effects of SSS on food intakes with adults [20]. That is, adults decrease their liking for the ad libitum consumed food, whereas the liking for the comparison foods which were not consumed ad libitum does not change (see [19,21] for reviews). Furthermore, it has been suggested that this decrease in liking is often the reason why people stop eating a particular food [20] and/or switch to other foods with a different sensory profile [22,23]. Meals that offer a greater variety of foods with different sensory characteristics (or sensations) then, may promote greater consumption due to a lack of SSS. Presently, though, little is known about SSS in children and particularly whether SSS occurs when children consume vegetables.

In providing children with a variety of foods, the effects of SSS may be reduced and therefore overall consumption increases. However, to date the effects of SSS on children’s liking and consumption of foods have been demonstrated twice, to the best of our knowledge, and neither of these studies used vegetables. In the first of these studies, Birch and Deysher (1986) gave twenty-one 2–5 year old children and 26 adults a variety of 99 g puddings to consume and concluded that SSS did take place in children [24]. Olsen and colleagues conducted a similar study with flavoured yoghurt (served at a maximum quantity of 750 g) and concluded that SSS in children was more product specific in children than in adults [25]. That is, children developed SSS only for the specific product they consumed, whereas adults also developed SSS for other products with a similar sensory profile as the consumed product, suggesting that SSS might differ in children and adults. It is not presently known whether SSS also occurs when children consume vegetables, although it is possible that it will differ from high energy dense foods, because low energy foods are usually less liked than high energy dense foods and therefore there is less of an opportunity to decrease in liking [16].

Studies with adults have also demonstrated the importance of the food’s sensory properties in affecting SSS: foods that require a lot of chewing (e.g., apples) generate a high level of oral stimulation, which results in high levels of SSS and consequently lower levels of food intake, compared to similar foods which require less chewing (e.g., apple sauce) [26,27,28]. Chewing and SSS can also be affected by the size of the offered food, in that small sized foods (i.e., foods that can be nibbled) are eaten at a slower pace than large sized foods which are eaten more quickly [29]. This slow eating pace is associated with a lower consumption, presumably due to a slow oral transit time. This results in a longer exposure to the sensory properties of food, which increases SSS [27]. Whether the same effects are seen with children is unknown. Children differ from adults in their food and taste preferences in a number of ways, for example they prefer higher levels of sweetness [30], saltiness [31,32] and to some extent sourness [33] and may therefore respond differently. For this reason, although it is helpful to draw on the existing adult literature on SSS, it is necessary to examine the effects of SSS specifically in children.

In the present study we aimed to explore the development of SSS as a result of children’s consumption of a fresh vegetable (carrots) in a diced and whole format. We hypothesized that SSS would occur when children consumed fresh carrots, and that SSS would be more likely to develop when children consumed diced, as opposed to whole carrots given that diced carrots can be nibbled and oral transit time may therefore be increased. We also hypothesized that SSS would affect carrot consumption, being lower in instances where SSS occurs.

## 2. Materials and Methods

### 2.1. Stimuli

Carrots and cucumber were selected as the test vegetables as they are reasonably well liked by children [34], are easy to manipulate in terms of size and have been used previously in sensory research with children [34,35,36,37,38,39]. Furthermore, fresh carrots and cucumber differ in their sensory profile from foods previously used in SSS experiments with children (yoghurts and puddings), as well as their nutrient profile (e.g., being much lower in energy density and protein) and therefore provide a useful counterpoint for testing whether SSS occurs with different food types. As the focus was on termination of vegetable consumption it was important to have a vegetable already liked by children to prevent immediate rejection upon first offering. For this reason carrots and cucumber were selected as the test vegetables. Cucumber served as a control food to determine the influence of ad libitum consumption on liking and was therefore only tasted and not consumed ad libitum. This is a standard way to assess SSS (see [19,20,26] for a review, see introduction, procedures and calculation for further explanation).

All vegetables were washed and prepared the day before testing and were presented in two sizes: diced and whole. Vegetables were cut in such a way that they could still be easily recognized. The diced vegetables were prepared as follows: carrots and cucumber were cut with the dimensions of approximately 2 × 2 × 2 cm per dice. The whole vegetables were prepared as follows: whole carrots consisted of the whole vegetable. A “whole” cucumber consisted of splitting a whole cucumber evenly in half and cutting them to 5 cm in length. All vegetables were refrigerated overnight in sealed containers and served fresh and uncooked. Carrots were served in carton popcorn boxes (20 cm in height, bottom: 7 × 7 × 7 cm, top: 9.5 × 9.5 × 9.5 cm) in large quantities (approximately 500 g per box) to minimize the likelihood that children would run out of carrots (mean weight: diced carrots = 460.4 ± 97.6 g and whole = 473.9 ± 88.6 g). The quantities of diced and whole carrots served were not significantly different (*p* = 1.0).

### 2.2. Measurement Tools

Children’s liking for carrots was measured with a 5-point hedonic scale with faces which represented 1 = really bad, 2 = bad, 3 = not bad nor good, 4 = good, or 5 = really good, which has successfully been used with similarly aged children [40]. Following Liem and Zandstra [40], the scales were clearly explained to the children, and to ensure that children understood the scale, each child was asked to point to the face they would make when they just ate something that tasted really good. Next children were asked to point to the face they would make when they just ate something which was neither bad nor good. All children were able to point to the correct face.

As food intake is also driven by participants’ hunger and satiety we also measured children’s perceived hunger before and after the ad libitum consumption of carrots. Under the supervision of a trained adult, children self-reported their hunger levels using an established methodology appropriate for use with primary school aged children [41,42]. The scale consisted of a visual representation (i.e., cartoon figures) of five levels of hunger varying from 1 = I do not feel full at all, I would eat a whole lot more; 2 = I feel a little bit full, I could eat more; 3 = I feel a bit full, I could eat a bit more; 4 = I feel quite full, I could eat a little bit more to 5 = I feel very full, I could not eat anything more (see [41] for more details). The hunger scale was also clearly explained to each child to ensure that it was understood. On each of the test days children were instructed not to eat or drink two hours prior to testing, although water was allowed.

### 2.3. Participants

Primary schools within a 30 km radius from Deakin University Burwood Campus (Melbourne, Australia) were invited by letter to take part in the study. The first two schools that consented to participate were selected and school staff discussed the study with the parents of children at their school. Those parents and teachers who expressed an interest in participating were sent a letter about the study via the school’s teachers. One week before the first test day, parents were sent two paper-and-pencil questionnaires containing questions on their child’s consumption of commonly eaten vegetables (data not reported here) as well as their demographic characteristics (e.g., child’s gender and age).

### 2.4. Procedure

The experiment included two test sessions spread out over two separate days, which were conducted at the child’s school. Testing on both days took place at the same time between 10 a.m. and 2 p.m. In a balanced order, children were presented with whole vegetables on one day and diced vegetables on the other day. Children were tested as part of a group, but each child had his/her own interviewer. Interviewers were trained beforehand and followed a predetermined script when conducting the hunger and liking tests with the children.

**Figure 1:**
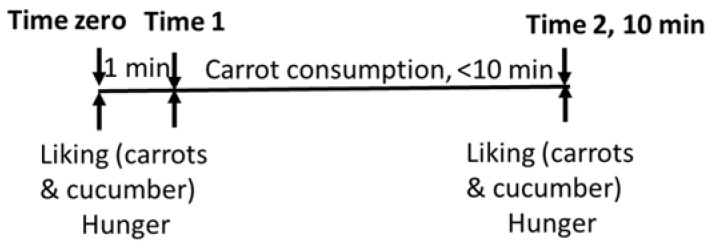
Schematic overview of the experimental session.

Figure 1 illustrates the flow of each session. During the test, children were given 10 min to eat as many carrots as they liked (time 1 to time 2 in Figure 1). Similar time frames were used in a previous study [34]. On each test day children started with rating their hunger levels, after which they were presented with carrots and cucumber in a balanced order (time zero in Figure 1). Children tasted and rated their liking of each of the vegetables after which all vegetables were removed from the table. Next, the ad libitum consumption took place: a box of 500 g of carrots was offered to each child with the instructions to eat as much or as little as they wanted (time 1). Children were instructed to keep the box in their lap, so it was always in sight. Children were asked to pay close attention to what they ate and were reminded to focus on their eating by the interviewers if necessary. When a child stopped eating prior to the 10 min testing limit, he or she was asked if he/she had finished eating. If the child said he or she had finished, the interviewer would ask again if he/she had finished eating. This continued until the child said he or she had finished eating on three consecutive occasions, after which the test was terminated. If the child still wanted to consume carrots after the time limit of 10 min lapsed, the researcher made a note of it and told the child that he/she had to stop eating. After the children stopped eating carrots, each rated their liking of carrots and cucumber (time 2). Children were not made aware that there was a time limit, however on the second day children might have expected that they were only allowed to eat carrots for 10 min. The box with carrots was weighed at time 1 and time 2.

All procedures were approved by the Deakin Human Ethics Committee.

### 2.5. Statistical Analyses

Statistical Package for the Social Sciences (SPSS Statistics version 19.0.0, IBM Corporation, New York, NY, USA) was utilized for the statistical analyses. A *p*-value of less than 0.05 was considered to be statistically significant.

SSS was defined as a change in liking of a food that has been consumed ad libitum (i.e., carrots) relative to a change in liking of a food that has not been consumed ad libitum (i.e., cucumber) [19]. In order to calculate SSS we followed the same statistical procedure as Weijzen et al. [26]. A change score for carrots was calculated by subtracting carrot liking after ad libitum consumption from carrot liking before ad libitum consumption, hereafter referred to as Change-Carrot-Score. A similar score was calculated for cucumber, hereafter referred to as Change-Cucumber-Score. The main Change-Carrot-Score was compared with the mean Change-Cucumber-Score by applying a Wilcoxon test. SSS was considered statistically significant if the mean Change-Carrot-Score was statistically significantly different from the mean Change-Cucumber-Score [26]. SSS was assessed for diced as well as whole carrots. Children’s carrot intake was calculated by computing the difference in weight of the popcorn boxes between before ad libitum consumption began (time 1) and time 2. Differences in perceived hunger and consumption between days during which children received the diced and the whole carrots were assessed by conducting a Wilcoxon analysis. Similar analyses were carried out to determine differences between boys and girls.

## 3. Results

### 3.1. Sample Characteristics

**Figure 2:**
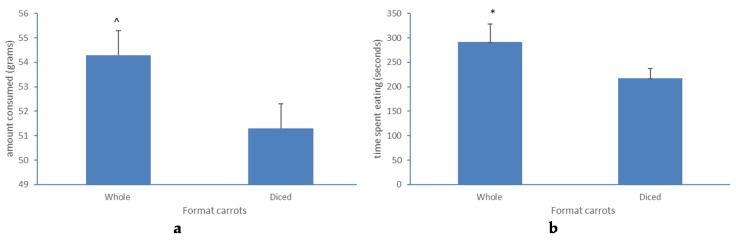
(**a**) panel-time (mean ± SEM) children spent eating carrots in seconds. (**b**) panel-amount children consumed (mean ± SEM) during 10 min. Shown for children who finished their consumption of whole and diced carrots before the 10 min time limit *n* = 36. * *p* < 0.05, ^ *p* = 0.06.

Seventy-two children (33 boys, ages 8.8 ± 1.6 years) participated (See Table 1). In general, children liked the taste of carrots at time zero, with no difference observed between diced and whole carrots (diced M = 4.4 ± 1.0, whole M = 4.4 ± 0.9). At time zero children reported to be moderately hungry, and perceived hunger was not different between test days (diced carrots M = 2.1 ± 1.1, whole carrots M = 2.4 ± 1.1, *p* = 0.08). At time 2 children had eaten M = 79.0 ± 55.1 g of the whole carrots (minimum = 1 g, maximum = 249 g) and M = 83.0 ± 74.6 g of the diced carrots (minimum = 1 g, maximum = 541 g). Five children consumed less than 5 grams of the whole carrots. Two children consumed less than 5 grams of the diced carrots. No statistical difference was observed between the consumption of whole and diced carrots (*p* = 0.95). Half (*n* = 36) of the children stopped eating carrots before they reached time 2. These children were significantly younger (*p* < 0.05), than those who did not stop eating before they reached time 2, and tended to eat more of the whole carrots (54.3 ± 89.0 g), than of the diced carrots (52.1 ± 45.8 g) (*p* = 0.06). The speed of eating the whole and the diced carrots was not different. However, children in this group spent more time eating whole carrots (291 ± 214 s) than diced carrots (216 ± 121 s) (*p* < 0.05) (see Figure 2). Hunger ratings between the days these children consumed whole and diced carrots was not different (diced carrots M = 2.0 ± 1.1, whole carrots M = 2.3 ± 1.2, *p* = 0.07). No association was found between perceived hunger at time zero and carrot consumption at time 2 (diced: *r* = −0.06, *p* = 0.63; whole: *r* = −0.017, *p* = 0.14).

### 3.2. Sensory Specific Satiety

Relative to liking of cucumber, liking for neither diced (*p* = 0.57), nor whole carrots (*p* = 0.18) changed between time 1 and time 2 (see Table 2) suggesting that SSS did not occur. When only including children who stopped eating carrots before the maximum time limit of 10 min was reached (*n* = 36), no evidence for SSS was apparent (diced carrots: *p* = 0.41; whole carrots: *p* = 0.25).

### 3.3. Liking and Consumption

Liking of whole carrots as measured at time zero was associated with intake (at time 2) of whole carrots (*r* = 0.25, *p* = 0.03). Liking of diced carrots was, however, not associated with consumption of diced carrots (*r* = 0.19, *p* = 0.12).

## 4. Discussion

The present study provides novel information on the development of SSS with consumption of a fresh vegetable and the role of food size. Although SSS has previously been observed in children with sweet dessert foods [38,39] our results showed that SSS did not occur with a fresh vegetable regardless of size. That is, children’s liking of carrots did not decrease between starting and terminating consumption in comparison to another fresh vegetable (cucumber). However, children, who finished eating within 10 min (mostly younger children), spent more time eating when presented with a whole carrot compared to diced carrots. This tended to result in an increased intake of the whole carrot compared to the diced carrot. These novel findings provide some preliminary evidence that despite the lack of SSS, the way vegetables are offered to children (i.e., diced or whole) might impact the amount they consume.

The present study demonstrated no decrease in liking between the start and termination of consumption of carrots in comparison to the test vegetable (cucumber). This study differed from those that have previously demonstrated SSS in children in its use of a fresh vegetable instead of sweet, high energy density foods, such as yoghurts or puddings [24,25]. The findings suggest that SSS occurs more slowly (or not at all) with a fresh vegetable than it may with foods higher in energy density and/or other sensory properties such as sweetness. This is despite the intense chewing and oral exposure involved in consuming a raw carrot [27,28,43] that prior research suggested may contribute to an increased likelihood of SSS [27,28,43]. SSS, then, appears not to be a reason for children terminating consumption of a fresh vegetable during one eating occasion like it may for dessert foods.

Studies investigating the effects of variety on children’s vegetable intakes have suggested that the lack of SSS might be one mechanism explaining greater vegetable intakes when variety is present [15]. However, the present findings do not support a role for SSS in preventing children’s greater vegetable consumption when lower variety is present. Furthermore, although SSS was not detected, half of the children in the present study stopped consuming carrots before the 10-min consumption period was complete, suggesting that other unmeasured factors were more important in affecting the amount of carrot consumed. Bergmaschi et al. [44] noted that children’s liking of and familiarity with particular vegetables was a more important determinant of their consumption than the variety of vegetables offered to 9–11 year old Danish school children. Other individual differences between children may also be important in determining SSS. Epstein et al. [16], for instance, noted that overweight children habituated more slowly to foods than children of healthy weight. Alternatively, it could be that children’s motivation to eat carrot diminished due to a lack of choice [45].

It is also possible that by terminating consumption before the allotted time period, children may not have ingested enough carrots to create SSS. During the consumption period children typically consumed 50–80 g of carrots, which is equivalent to about one medium sized carrot, or just over one serving (i.e., 75 g). This is close to Australian children’s typical daily vegetable consumption [7]. In previous SSS experiments with children, subjects consumed more servings (e.g., Olsen’s participants consumed seven servings of yoghurt [25]. It is possible, then, that the children in the present study did not consume enough carrots for SSS to occur. However, studies of children’s vegetable consumption have noted that children are unlikely to consume more than 100 g of vegetables per day [15,44], suggesting that it is unlikely that children will consume more than this in a short time period. Future studies could consider exploring the amount of different foods required before SSS does occur, especially for low energy dense foods but allowing children a greater period of time for consumption, for instance.

Interactions with other factors such as the food’s sensory properties [11,44,46,47] and the nutrient profile or energy density of the test food (along with children’s liking and familiarity) may confound results. In adults, there is some evidence that a food’s macronutrient composition may affect SSS (see [21] for review). Similarly, it has been suggested that foods high in energy density show a greater level of SSS than low energy dense foods [48,49], whereas others failed to see such relationship [24,50]. To date, the influence of a food’s macronutrient composition and energy on SSS in children is largely unknown. Olsen et al. [25] observed SSS in children when using high energy and high protein foods. We observed no SSS with carrots which contained half the energy and one third of the protein content compared to the food used by Olsen et al. [25], but the foods also differed in other variables such as the sensory properties.

In the present study we manipulated the food’s size, hypothesizing that this would be a factor influencing the development of SSS due to a greater oral processing time. However, the results demonstrated that size (whole or diced carrot) did not affect SSS. Children who finished their carrot consumption before the allotted time of 10 min spent, however, more time eating the whole carrots, which tended to result in a higher intake compared to diced carrots. This is in line with previous studies which found that large unit sizes increased food consumption [26,51,52]. It is important to note that children in the present study did not eat whole carrots quicker than diced carrots. They simply spent more time eating. This suggests that the observed difference is unlikely due to a difference in oral transit time. Potentially the results can be explained by unit-bias [53], in which a given unit creates a consumption norm, which tells consumers how much they should eat. Children in the present study consumed about one whole carrot (one unit) when presented with whole carrots, suggesting that once children started eating a whole carrot they were likely to finish it. It is also important to note that children who finished before the 10 min time limit, were younger than those who wanted to continue eating. The unit-bias effect as observed with the younger children might also apply to the older children if they were given more time to consume carrots. To our knowledge this is the first study with children which suggest that unit bias can be used to increase consumption of healthy foods.

In summary, it is possible that SSS does not occur when children consume a fresh vegetable. However, the reasons underlying the lack of SSS with a fresh vegetable are unknown, as the present study was not designed in a way to enable us to tease out the contributions of a range of possible factors that may affect the development of SSS in the present study. Future research on SSS in children would benefit from using a variety of products that systematically differ in macronutrient composition, energy density, sensory profiles (e.g., sweetness, fattiness) and test the effects of these amongst children who differ in their initial liking of the foods and other characteristics such as their weight status to tease out the effects of each of these. Allowing children a greater period of time to consume the test food may also be beneficial. This research would provide useful insights into how and why SSS develops when children consume health promoting foods such as vegetables. Despite the lack of SSS, this study points out that by proving vegetables in a larger unit size consumption can be increased.

The present study presents a novel approach to examining the challenge of increasing children’s vegetable intakes. However, it is subject to limitations such as those described earlier including a design that does not enable the contributions of a range of factors on the development of SSS to be examined. A standard methodology was employed here which has been able to detect SSS previously [54] and the significant association between carrot liking and carrot consumption attests to the validity of the current tools to accurately measure children’s liking. Furthermore, the study was also adequately powered based on previous SSS studies [24,25] and should therefore have the capacity to detect SSS should it occur. Future studies testing the effects of SSS with repeated tastings, and/or with a number of different vegetables would provide further information on the development of SSS when children consume vegetables.

## 5. Conclusions

This work provides an insight into the effects of SSS and unit size on children’s consumption of a fresh vegetable. The findings indicated that, using a standard SSS experiment, SSS was not observed when children consumed fresh whole or diced carrots and therefore SSS did not pose a barrier to their consumption. The role of SSS in affecting the variety of vegetables children consume in a single eating occasion therefore remains uncertain. A larger unit size can, however, be beneficial to children’s vegetable consumption. Future studies designed in ways to tease out the effects of the food’s unique sensory and nutrient profile on SSS in children are needed. Such studies will aid in the development of strategies to promote greater consumption of vegetables by children within one eating occasion.

## Figures and Tables

**Table 1:** Characteristics of the participants in the experiment.

Subjects		Sample	
	**Total**	**Did Child Finish Consumption? ^1^**	
		**Yes**	**No**
Children			
*N*	72	36	36
Boys	33	16	17
Age (years)	8.8 ± 1.5	8.1 ± 1.2	9.5 ± 1.7 *
BMI ^2^	17.6 ± 3.5	17.0 ± 2.9	18.2 ± 4.1
Parent’s marital status			
Single	8%		
De facto/married	81%		
Divorced	3%		
Missing	2%		
Parent’s education (highest completed degree)			
High School	32%		
TAFE ^3^	18%		
University degree	50%		

**^1^** Did child finish consumption (before the 10 min time limit)? **^2^** BMI = Body Mass Index (kg/m^2^); **^3^** TAFE = Technical and Further Education; * *p* < 0.05.

**Table 2:** Liking of carrots and cucumber (whole and diced) at time zero and time 2.

	Time 0	Time 2
	Liking	Liking
Carrots		
Whole	4.4 ± 0.9 ^1^	4.5 ± 1.0
Diced	4.4 ± 1.0	4.4 ± 0.8
Cucumber		
Whole	4.1 ± 1.3	4.2 ± 1.2
Diced	4.3 ± 1.0	4.3 ± 1.1

**^1^** Mean ± SD (standard deviation), measured on a 5-point hedonic scale.
